# VMAT testing for an Elekta accelerator

**DOI:** 10.1120/jacmp.v13i2.3725

**Published:** 2012-03-08

**Authors:** Darryl G.L. Kaurin, Larry E. Sweeney, Edward I. Marshall, Saikanth Mahendra

**Affiliations:** ^1^ Northwest Medical Physics Center Lynnwood WA; ^2^ Seattle Cancer Care Alliance – Radiation Oncology Seattle WA USA

**Keywords:** VMAT commissioning, IMAT commissioning, linac quality a ssurance, DMLC

## Abstract

Volumetric‐modulated arc therapy (VMAT) has been shown to be able to deliver plans equivalent to intensity‐modulated radiation therapy (IMRT) in a fraction of the treatment time. This improvement is important for patient immobilization/ localization compliance due to comfort and treatment duration, as well as patient throughput. Previous authors have suggested commissioning methods for this modality. Here, we extend the methods reported for the Varian RapidArc system (which tested individual system components) to the Elekta linear accelerator, using custom files built using the Elekta iComCAT software. We also extend the method reported for VMAT commissioning of the Elekta accelerator by verifying maximum values of parameters (gantry speed, multileaf collimator (MLC) speed, and backup jaw speed), investigating: 1) beam profiles as a function of dose rate during an arc, 2) over/under dosing due to MLC reversals, and 3) over/under dosing at changing dose rate junctions. Equations for construction of the iComCAT files are given. Results indicate that the beam profile for lower dose rates varies less than 3% from that of the maximum dose rate, with no difference during an arc. The gantry, MLC, and backup jaw maximum speed are internally consistent. The monitor unit chamber is stable over the MUs and gantry movement conditions expected. MLC movement and position during VMAT delivery are within IMRT tolerances. Dose rate, gantry speed, and MLC speed are accurately controlled. Over/under dosing at junctions of MLC reversals or dose rate changes are within clinical acceptability.

PACS numbers: 87.55.de, 87.55.Qr, 87.56.bd

## I. INTRODUCTION

Volumetric‐modulated arc therapy (VMAT) is being used to deliver intensity‐modulated radiation therapy (IMRT) treatments with shorter treatment duration for each fraction and, in some cases, a better dosimetric plan.^(^ ^1^ ^,^ ^2^ ^,^ ^3^ ^,^ ^4^ ^)^ A shorter treatment time is a significant improvement for both patient compliance issues, due to confining immobilization and respiratory control devices, and patient throughput. While IMRT arc therapy is not new,^(5)^ VMAT's recent wide‐spread availability with accelerators having standard multileaf collimators (MLCs) (40cm×40cm) is due to delivery and planning systems that can now modulate and monitor gantry speed, dose rate, and MLC position simultaneously. To verify that the linear accelerator (linac) can correctly deliver a VMAT treatment, Ling et al.^(6)^ suggested commissioning guidelines for RapidArc commissioning (Varian Medical Systems, Palo Alto, CA), and downloads of the accelerator controller files for the commissioning are available for physicists. Bedford and Warrington^(7)^ suggested guidelines for VMAT commissioning in general, and used a different vendor's linac (Elekta Ltd, Crawley, UK). Their tests, while helpful for Elekta customers by investigating parameters specific to Elekta (e.g., dose rate, in particular), do not test the individual components of the treatment modality directly, as Ling et al.^(6)^ did (e.g., multicollimator leaf control, gantry speed). Most Elekta VMAT physicists have commissioned their systems by comparing measured dose profiles of VMAT‐planned patient cases. While this is a necessary component for commissioning a system, we thought this to be inadequate for catching errors that would be noticed by measuring individual components. (For example, a Picket Fence MLC test will catch 1 to 2 mm errors that will not be seen in patient specific IMRT QA). We present here the methods used by Ling et. al.,^(6)^ augmented with several of the tests suggested by Bedford and Warrington^(7)^ pertinent for an Elekta LINAC. The differences between our approach and that of Ling et al. is that the controlling VMAT files were constructed in‐house for an Elekta linac, with additional tests specific to our case. The differences between our approach and that of Bedford and Warrington is an extension of some of their tests, excluding reliance on the treatment planning system (TPS), as well as additional tests (open beam profiles of differing dose rates during arcs, fluence errors during rapid MLC reversals, fluence errors at dose rate change junctions, verifying internal consistency of maximum gantry, MLC speed, and jaw speeds, and covering a range of gantry and MLC speeds up to and exceeding the maximum values). The TPS with end‐to‐end testing is necessary for commissioning, but not investigated here.

## II. MATERIALS AND METHODS

The accelerator tested for this project was an Elekta Infinity with the MLCi2. The MLC has 40 leaf pairs, each with a width of 1 cm at isocenter, a minimum gap of 0.5 cm at isocenter; the MLC is interdigitation‐capable (software necessary for interdigitation is waiting regulatory approval). Both 6 and 10 MV will be used for VMAT delivery. Tests with no dose rate (DR) considerations were carried out for 6 MV only. We constructed VMAT files controlling each component of the delivery using the iCom Customer Acceptance Test (iComCAT) version 1.0.0.13 (Elekta Ltd, Crawley, UK). Care must be exercised using this program in accordance with the caution noted: the software “lacks safeguards normally present in a program intended to prepare and deliver clinical prescriptions”.

### A. General linac characteristics

#### A.1 Detector positioning accuracy

##### A.1.1 Imager center pixel test

The electronic portal imaging device (EPID) was used for the MLC positioning (Picket Fence) and other tests. Imager sag was characterized by taking images using the EPID of open fields with an open‐air graticule (Aktina Medical Corporation, Congers, NY) in place. The image analysis software used for the central BB locations, as well as other EPID analysis, was ImageJ (available at http://imagej.nih.gov).

#### A.1.2 MapCHECK2 geometric accuracy

The MapCHECK2 was used for dosimetric measurements. It was mounted to the gantry using the Isocentric Mounting Fixture (IMF) (SunNuclear Corp, Melborne, FL) with a 3 cm buildup plate to give an effective depth of 5 cm (the device has 2 cm of inherent buildup). Diodes used for the measurements were in areas designed to achieve uniform dose, so geometric accuracy is not as critical as the EPID, but nevertheless needed to be quantified. This was measured by mounting the device to the IMF without buildup and measuring the distance of the light‐projected crosshair to the center mark of the device. Since the sag might be different with the 3 cm buildup plate, the plate was placed on the IMF at each gantry position without obscuring the crosshair.

#### A.2 Dosimetric tests

For brevity and consistency with others, we use the following in equations and when identifying particular values of each parameter:^(3)^

θ=gantryangle; Δθ/Δt=gantryspeed; x=positionofanMLCleaf; Δx/Δt=leafspeed; MU=doseinmonitorunits; ΔMU/Δt=doserate; Δθ/ΔMU=gantryangletraversalperMU; max=maximum; min=minimum.

Also for brevity and reading ease, we use additional abbreviations for dose rate (DR) and gantry speed (GS).

Dosimetric tests for general linac performance were carried out by positioning an ionization chamber with a buildup cap at isocenter. Charge was measured using an electrometer with high‐charge collection capability. These measurements are an extension of the work by Bedford and Warrington,^(7)^ and have been used by others for dynamic MLCs (DMLC)^(8)^ and RapidArc commissioning.

##### A.2.1 Arc dosimetry tests

The monitor unit chamber stability was tested over the range of expected VMAT MUs for both static and 360° arc fields using a 10cm×10cm field with the ion chamber placed at 100 cm source‐to‐axis distance for both 6 and 10 MV beam. Monitor unit values were 36 and 1000 MUs with DR values of ${\left(\Delta \text{MU}/\Delta t\right)}_{\min}$ and ${\left(\Delta \text{MU}/\Delta t\right)}_{\max}$, respectively. The 36 MU value corresponds to ${\left(\Delta \theta /\Delta \text{MU}\right)}_{\min}$. Comparisons were made between static and arc fields, and linearity between the two dose levels was calculated.

##### A.2.2 DMLC tests

Gravity effects on a dynamic treatment were investigated by constructing a 23cm×10cm field with a dynamic moving gap of 1 cm with the gantry at cardinal angles and a 180° arc (gantry vertically pointing up to pointing down) and measured using a Farmer‐type ionization chamber with a buildup cap with the cylinder barrel oriented perpendicular to the MLC motion direction.^(^ ^6^ ^,^ ^7^ ^,^ ^8^ ^)^ The collimator angle was oriented to have the maximum gravity effect on the MLC movement (movement perpendicular to the ground for a horizontal gantry). (Additional field parameters: 6 MV, DR: maximum, MLC speed=0.74cm/sec along the 23 cm width, arc gantry $\text{speed}=5.8^{\circ }/\sec$.) Comparisons between each measurement with the average static measurement were carried out.

##### A.2.3 Flatness and symmetry as a function of dose rate

Bedford and Warrington^(7)^ showed flatness and symmetry varied slightly as a function of DR in a water tank. Like their linac, our dose rates are binned as multiples of 2 from the maximum dose rate. We were concerned that the flatness and symmetry as a function of DR might not be independent of gantry angle during an arc, so we measured this using a MapCHECK2 mounted to the gantry using the Isocenteric Mounting Fixture for a 25cm×25cm field at a vertical and horizontal angle, as well as during a 358° arc, which was thought to be sufficient to identify any problems. The absolute MUs delivered for the arc case with different DRs were changed for each arc to maintain a gantry speed between 4° and 4.5°/sec, except for the lowest dose rate which had a gantry speed of about 3°/sec (necessary so the maximum gantry speed was not exceeded). The depth of measurement was 5 cm equivalent for both 6 and 10 MV. The dose rates we show are nominal to compare with Bedford and Warrington;^(7)^ the absolute maximum dose rate between Elekta accelerators varies slightly, depending on tuning. We also extend the Bedford and Warrington data to include ${\left(\Delta \text{MU}/\Delta t\right)}_{\max}/32$, which we do see occasionally during VMAT treatments. Note that the beam profile measurements for each DR for the vertical gantry were used for normalization of subsequent tests (see below). An EPID or film can also be used for these measurements, but the 2D diode array is sufficient in the authors' opinions, especially considering the convenience and ease‐of‐use. We encountered pixilation effects with the EPID for several of the tests in the low DR region that made the 2D diode array more attractive.

### B. MLC positional tests

Dynamic Picket Fence patterns^(9)^ were constructed for a field size of 25cm×25cm; the EPID was used to acquire the dosimetric‐positional image.

#### B.1 Picket Fence test for static and arcing gantry

For these tests, a Picket Fence with a moving gap of 5 mm was used, except at desired positions a 1 mm strip was enhanced by advancing the leading MLC 1 mm and pausing the trailing MLC over the 1 mm strip; but either or both MLCs are moving continuously. The collimator angle was oriented to have the maximum gravity effect on the MLC movement (movement perpendicular to the ground for a horizontal gantry). The Picket Fence test was run at the cardinal angles and a 358° arc. EPID image data was captured using ImageJ and imported into a spreadsheet to quantitatively analyze the Picket positions.

#### B.2 Picket Fence test with intentional errors

As with Ling et al.,^(6)^ intentional errors were introduced to ensure that unknown errors could be identified. A Picket Fence with intentional errors during an arc having one leaf pair with a 1.5 mm wide strip instead of the 1 mm strip was constructed, and another leaf pair having a 0.5 mm positional shift. The gap for this DMLC was 6 mm, to allow for the errors, as the adjacent leaves would be closer than the minimum 5 mm gap. This was analyzed as above.

### C. VMAT tests

For the Elekta linac, maximum values for gantry speed, MLC speed, backup jaw speed, and collimator rotation speed are input into the software and are readily displayed. TPSs currently do not allow for dynamic collimator rotation, so this was not tested. Rather than timing these values absolutely, which introduces subjectivity due to ramp‐up speed, we chose to verify these for internal consistency using the following tests.

#### C.1 Verifying DR, GS, leaf speed, and backup jaw speed

Presently, ΔMU/Δt for our Elekta accelerator is binned as multiples of half of the maximum DR, which are displayed. We used the ${\text{DR}}_{\max}$ to determine the GS and MLC speed. To determine the accuracy of the displayed ${\text{DR}}_{\max}$, 500 MUs were delivered for both 6 and 10 MV and timed with a stop watch. The time needed for the average ${\text{DR}}_{\max}$ displayed was compared with the measurement.

##### C 1.1 MLC speed

A maximum MLC speed of 2 cm/sec is recommended by the vendor and was entered in the controlling software. This was tested for internal accuracy by constructing a 10cm×10cm field using a DMLC with an 8 mm gap (which we use clinically) in the iComCat software. The MUs for this field are calculated using Eq. (1):

(1) $$MU=\frac{{\left(\Delta \text{MU}/\Delta t\right)}_{\max}(x_{f}-x_{i})}{{\left(\Delta x/\Delta t\right)}_{\max}}$$

where $\left(x_{f}-x_{i}\right)$ is the final and initial MLC position, which was 10 cm for our test.

The calculated MUs and other field parameters were input into an iComCat file and executed. If the displayed DR is maximum, the MLC speed may be greater than 2 cm/sec, and the MUs can be incrementally decreased until the DR drops to half‐maximum. The actual MLC speed can then be calculated by solving Eq. (1) for ${\left(\Delta x/\Delta t\right)}_{\max}$. Similarly if the displayed DR is half‐maximum, the maximum MLC speed is less than the nominal speed; the MUs can be incrementally increased for the field until they run at ${\left(\Delta \text{MU}/\Delta t\right)}_{\max}$, at which point the MLCs will be at the maximum speed. Fractional MU entry is allowed, so MLC speed within 0.1 cm can be calculated.

##### C.1.2 Backup jaw speed

The maximum backup jaw speed in the controlling software was 1.5 cm/sec. The same method as given in C.1.1 for the MLC speed was used to verify internal consistency of this value.

##### C.1.3 Maximum gantry speed

The gantry speed given by the vendor was 6.0°/sec which was input into the controlling software. The MUs for the verification measurement were calculated using:

(2) $$MU=\frac{{\left(\Delta \text{MU}/\Delta t\right)}_{\max}(\theta_{f}-\theta_{i})}{{\left(\Delta \theta /\Delta t\right)}_{\max}}$$

where $\left(\theta_{f}-\theta_{i}\right)$ are the final and initial gantry positions, which was 180° for our test, and ${\left(\Delta \theta /\Delta t\right)}_{\max}$ was the nominal maximum given by the vendor.

The parameters for the nominal gantry speed from Eq. (2) were inputted into an iComCAT file. As with the MLC speed determination, if the field was delivered and the displayed DR was maximum, the MUs were decreased until the DR dropped by half. The maximum gantry speed can then be calculated by solving Eq. (2) for gantry speed using the minimum MUs that resulted in the maximum DR delivery. If the nominal values resulted in the DR not being maximum, the gantry speed is slower than the nominal value, and must be increased until the delivered DR is maximum, and then one must solve Eq. (2) again for ${\left(\Delta \theta /\Delta t\right)}_{\max}$.

#### C.2 Verifying dynamic delivery

##### C.2.1 Verify DR and GS

To verify accurate DR and GS during VMAT delivery, a test with eight horizontal strip combinations of DR and GS was constructed where strips had DR:GS values of: ${\left(\Delta \text{MU}/\Delta t\right)}_{\max}/32:{\left(\Delta \theta /\Delta t\right)}_{\max}/32$; ${\left(\Delta \text{MU}/\Delta t\right)}_{\max}/16:{\left(\Delta \theta /\Delta t\right)}_{\max}/16$; ${\left(\Delta \text{MU}/\Delta t\right)}_{\max}/8:{\left(\Delta \theta /\Delta t\right)}_{\max}/8$; ${\left(\Delta \text{MU}/\Delta t\right)}_{\max}/4:{\left(\Delta \theta /\Delta t\right)}_{\max}/4$; ${\left(\Delta \text{MU}/\Delta t\right)}_{\max}/2:{\left(\Delta \theta /\Delta t\right)}_{\max}/2$; ${\left(\Delta \text{MU}/\Delta t\right)}_{\max}:{\left(\Delta \theta /\Delta t\right)}_{\max}$; $1.2{\left(\Delta \text{MU}/\Delta t\right)}_{\max}:{\left(\Delta \theta /\Delta t\right)}_{\max}$; ${\left(\Delta \text{MU}/\Delta t\right)}_{\max}:1.2{\left(\Delta \theta /\Delta t\right)}_{\max}$. Note the last two strips have either the DR or GS greater than the maximum, to determine if the dose is still correctly delivered.

The MUs delivered in each strip and moving between strips were constant to allow relative analysis, as suggested by Ling et al.^(6)^ We wanted the MUs delivered while moving between the strips to be a minor contribution of the strip dose. We also wanted the MLC speed in moving between strips to be constant to minimize this variable for this test, which requires the DR moving between strips be constant. We also wanted the GS moving between the strips to be the same as the previous strip.

These conditions were met by first calculating the time taken for the gantry arc between strips:

(3) $$t_{i\to j}=\frac{x_{i\to j}}{{\left(\Delta x/\Delta t\right)}_{i\to j}}$$

where $t_{i\to j}=$ the time taken in moving between strips, and $x_{i\to j}$ is the width of the strips. For this test, we chose ${\left(\Delta x/\Delta t\right)}_{i\to j}=0.75{\left(\Delta x/\Delta t\right)}_{\max}$.

Then:

(4) $${\text{MU}}_{i\to j}=t_{i\to j}{\left(\Delta MU/\Delta t\right)}_{i\to j}$$

where ${\text{MU}}_{i\to j}$ are the MUs moving between strips. In selecting ${\left(\Delta \text{MU}/\Delta t\right)}_{i\to j}$, the physical constraint $\Delta \text{MU}/\Delta \theta \geq 0.1\text{MU}{/}^{\circ }$ cannot be violated. This is verified using:

(5) $$(\Delta \text{MU}/\Delta \theta )=\frac{{\left(\Delta \text{MU}/\Delta t\right)}_{i\to j}}{{\left(\Delta \theta /\Delta t\right)}_{\max}}\geq 0.1\text{MU}/{}^{\circ}$$

${\left(\Delta \text{MU}/\Delta t\right)}_{i\to j}$ was chosen to be ${\left(\Delta \text{MU}/\Delta t\right)}_{\max}/8$ so the DR ramp up/down would not be too extreme for the slower or faster DRs in each strip.

Except for the strips with 1.2 times DR or GS, the total angle traversed is the same for each strip, and is calculated as:

(6) $$\theta_{i}=\frac{{\text{MUs}}_{\text{stip}\;\text{constant}}{\left(\Delta \theta /\Delta t\right)}_{i}}{{\left(\Delta \text{MU}/\Delta t\right)}_{i}}$$

where $\theta_{i}$ is the angle for the strip i; and where ${\text{MU}}_{\text{stripconstant}}$ were the MUs chosen to be significantly larger than the MUs delivered when moving from one strip to the next, but restricted to a value such that the sum of the gantry angles traversed for all strips and movement between strips is less than a complete arc, which can be calculated, but we solved it iteratively using a spreadsheet.

The strips were 3 cm wide with initial and ending segments of 0.5 cm. The measurement point was at the middle of each strip. The measurement device was the same used for the open beam profiles (above). The field sizes and measurement depth for the beam profiles and these measurements were the same, which allowed us to use the beam profiles to normalize the measurements here for the off‐axis ratio. The measurement points were at the middle of each strip, and the middle of the MLC leaf pair to minimize interleaf leakage considerations. To minimize the effect of a single aberrant diode measurement, 12 diodes in the strip were averaged (central 11 cm of the strip). Each diode measurement was normalized using the open field with the same DR at the same detector position.

##### C.2.2 Test MLC speed

To test accurate control of MLC speed during VMAT delivery, an iComCAT file was constructed having a moving gap of 0.8 mm (used clinically), constant GS, and strips having differing MLC speed and DR (MLC speed: DR): ${\left(\Delta x/\Delta t\right)}_{\max}/32:{\left(\Delta \text{MU}/\Delta t\right)}_{\max}/32$; ${\left(\Delta x/\Delta t\right)}_{\max}/16:{\left(\Delta \text{MU}/\Delta t\right)}_{\max}/16$; ${\left(\Delta x/\Delta t\right)}_{\max}/8:{\left(\Delta \text{MU}/\Delta t\right)}_{\max}/8$; ${\left(\Delta x/\Delta t\right)}_{\max}/4:{\left(\Delta \text{MU}/\Delta t\right)}_{\max}/4$; ${\left(\Delta x/\Delta t\right)}_{\max}/2:{\left(\Delta \text{MU}/\Delta t\right)}_{\max}/2$; ${\left(\Delta x/\Delta t\right)}_{\max}:{\left(\Delta \text{MU}/\Delta t\right)}_{\max}$; $1.2{\left(\Delta x/\Delta t\right)}_{\max}:1.2{\left(\Delta \text{MU}/\Delta t\right)}_{\max}$. For the dose per strip to be constant for ease of analysis, the MUs were determined using:

(7) $$MU=\frac{(x_{f}-x_{i}){\left(\Delta \text{MU}/\Delta t\right)}_{\max}}{{\left(\Delta x/\Delta t\right)}_{\max}}$$

where $\left(x_{f}-x_{i}\right)$ are the final and initial MLC positions for each strip (3 cm width for each strip). The input GS was constant for this test, and was calculated using Eq. (5) for 0.1MU/° with ${\left(\Delta \text{MU}/\Delta t\right)}_{i\to j}={\left(\Delta \text{MU}/\Delta t\right)}_{\max}/32$. The actual deliverable gantry speed for the last strip with $1.2{\left(\Delta x/\Delta t\right)}_{\max}$ would be less by a factor of 1.2, since the MLC can't be driven faster than the maximum.

Strips of 1.5 cm width on the lateral sides of the field were also irradiated to the same dose so the field size would be the same as the open fields used in Section A.2.3 above, to enable normalization of the test field. Only 6 MV was used for the test, since it is an MLC speed test. The same measurement and analysis techniqueswere used as with the DR and GS test.

As a cautionary note, we initially designed this test with the minimum gap of 0.5 cm, which gave poor results at the maximum MLC speed (actively displayed as well as dosimetrically). Since these tests are outside of clinical mode which may have interrupted the treatment, this gap width using this software is not suggested when driving the MLC near maximum speed.

##### C.2.3 MLC reversals

**Figure 1:**
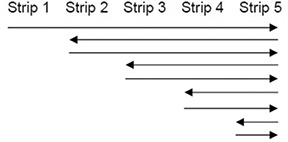
Schematic for investigating the effect of rapid MLC reversals. Arrows indicate MLC travel direction.

Bedford and Warrington^(7)^ investigated if the MLCs could reverse direction accurately via a treatment plan, which was not considered by Ling et al.^(6)^ We investigated this by making an iComCat file with five 4 cm wide strips with dose rates ${\left(\Delta \text{MU}/\Delta t\right)}_{\max}/2$ with differing numbers of traversals (schematically shown in Fig. 1) at MLC speeds of 1.0, 0.95, 0.90, and 0.18 ${\left(\Delta x/\Delta t\right)}_{\max}$. There are single reversals between the first four strips, and 4.5 reversals on the far right strip. The dose in each strip increases with the number of traversals. The EPID was used for the fluence measurement to obtain a continuous MLC movement profile and ImageJ was used for analysis. Individual pixel data at the junction changes was erratic, so the image data was smoothed manually by using a moving three‐pixel average (i.e., the pixel itself with the proceeding and succeeding pixel; each pixel is 0.256 mm, so the average is over 0.77 mm). Smoothing was not thought to mask clinically significant effects occurring over 0.5 mm. The 1.0, 0.95, 0.90 ${\left(\Delta x/\Delta t\right)}_{\max}$ measurements were normalized using the 0.18 ${\left(\Delta x/\Delta t\right)}_{\max}$ measurement, which results in a relative analysis of uniformity in the ideal case of no over‐travel. The reference measurement corresponds to 0.36 cm/sec which was chosen by default using the maximum number of MUs for imaging (999 MU), and is assumed to have negligible over‐travel. The DR for the test ${\left(\Delta \text{MU}/\Delta t\right)}_{\max}/2$ was chosen over the maximum to obtain the slower MLC speed for the reference measurement while providing adequate signal.

##### C.2.4 Dose‐rate changes

The effect of DR changes was investigated with other factors in previous sections using measurements at the middle of each strip. We wanted to also look at instantaneous DR increases and decreases between strips. The DR increase test used eight 3 cm wide strips with DRs increasing from ${\left(\Delta \text{MU}/\Delta t\right)}_{\max}/32$ to ${\left(\Delta \text{MU}/\Delta t\right)}_{\max}$, sequentially by factors of 2, with the last two strips testing the extreme case of the dose increasing from ${\left(\Delta \text{MU}/\Delta t\right)}_{\max}/32$ to ${\left(\Delta \text{MU}/\Delta t\right)}_{\max}$. The MLC speed needed to be constant to investigate the DR alone; we were concerned that the maximum MLC speed may not give enough dose for an adequate signal, so ${\left(\Delta x/\Delta t\right)}_{\max}/2$ was used for the test. The fluence in each strip was in proportion to the DR for the strip. The gantry speed was constant for the test, with the speed calculated using Eq. (5) setting the equation to equal 0.1 MU/°, using ${\left(\Delta \text{MU}/\Delta t\right)}_{\max}/32$ for ${\left(\Delta \text{MU}/\Delta t\right)}_{i\to j}$ and solving for (Δθ/Δt). As with the MLC rapid‐reversals test, the fluences were measured using the EPID to get a continuous profile, and the test measurement values were normalized for unity analysis using a reference measurement having all parameters the same except using a MLC speed of 0.25 cm/sec. Two measurements were made for both the test and reference irradiations, and were averaged pixel by pixel, again due to our concerns of the dose being too low for an adequate signal. Both energies used for VMAT, 6 MV and 10 MV, were tested.

The effect of DR decrease was investigated as with DR increase, except the DRs decrease from ${\left(\Delta \text{MU}/\Delta t\right)}_{\max}$ to ${\left(\Delta \text{MU}/\Delta t\right)}_{\max}/32$ in factors of 2 as the MLC bank moves from left to right. Unlike the DR increase test, there was some vertical pixilation in the images. Since we had two images for each measurement, any pixilation points (identified during analysis by excursions of >3% relative to pixels 0.5 mm on either side) were deleted with the average between the two measurements including just the unpixilated portion. Deletion of vertical pixilation data was easily verified manually, since the profiles showed the single data spikes, and there were less than eight lines in each image.

## III. RESULTS

### A. Detector positioning accuracy

#### A.1 Imager center pixel test

The deviation of the central ray on the panel is well within ±1 mm in the cross‐plane direction (parallel to the direction the MLCs move in) (Table 1). Therefore, any sag effects are negligible and the panel can be used for subsequent tests. There is some deviation in the in‐plane direction of ±1 mm, but this is still within acceptable limits, and will not affect the results here, as the MLCs move in the cross‐plane direction.

**Table 1:** Linac central axis projected on EPID. X and Y refer to cross‐ and in‐plane distance, respectively.

*Gantry Angle*	*X Center Distance (cm)*	*Y Center Distance (cm)*	*X Distance From Ave (cm)*	*Y Distance From Ave (cm)*
180	13.06	13.37	0.005	0.103
90	13.06	13.27	0.005	0.003
270	13.04	13.27	−0.015	0.003
0	13.06	13.16	0.005	−0.107

#### A.2 MapCHECK2 geometric accuracy

The deviation of the center mark of the MapCHECK2 attached to the gantry using the IMF at the cardinal angles measured using the field light crosshair was within ±0.5mm. This was thought to be adequate, as the tests using the device were designed to deliver uniform dose over distances >5mm on either side of the diodes used.

#### A.3 Arc dosimetry tests

##### A.3.1 Monitor chamber stability

Ion chamber readings for both static and arc gantry treatments with a static field were within 0.6% for both 6 and 10 MV (Table 2). Linearity for the static 36 and 1000 MU tests were within 0.3%.

**Table 2:** Monitor chamber stability for static gantry and clockwise and counterclockwise 360° arc fields.

*6 MV*					*10 MV*				
*Static*			*360° Arc*		*Static*			*360° Arc*	
36 MU Reading	1000 MU Reading		36 MU Reading	1000 MU Reading	36 MU Reading	1000 MU Reading		36 MU Reading	1000 MU Reading
(nC)	(nC)		(nC)	(nC)	(nC)	(nC)		(nC)	(nC)
6.361	176.70	CW	6.325	176.94	5.999	166.26	CW	5.962	166.23
		CCW	6.324	176.82			CCW	5.965	166.12
Arc/Static:			0.994	1.001				0.994	0.999

##### A.3.2 DMLC tests

Ion chamber readings of the DMLC gap at all cardinal angles and the arc delivery were within ±0.5% with respect to the average static reading (Table 3). This indicates negligible gravity effect on DMLC fields.

**Table 3:** Ion chamber measurements of gravitational effects on DMLC delivery for a 1 ‐cm moving gap. The 180° arc is from vertical up to vertical down.

*Gantry Angle*	*Reading (nC)*	*Ratio with Static Average*
0	2.09	1.001
90	2.08	0.996
180	2.09	1.001
270	2.09	1.001
180°Arc	2.08	0.996

##### A.3.3 Flatness and symmetry as a function of dose rate

**Figure 2:**
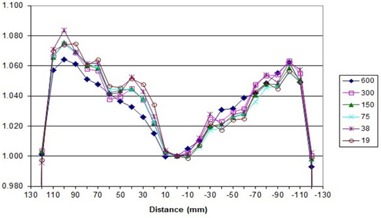
Crossbeam profile for vertical gantry angle for all DRs for 6 MV.

**Figure 3:**
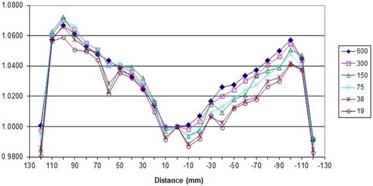
Crossbeam profile for 358° arc for all DRs for 6 MV

**Figure 4:**
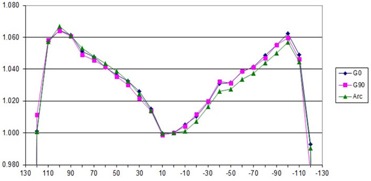
Crossbeam profile for vertical and horizontal gantry angles and 358° arc for 600 MU/min DR for 6 MV.

To avoid confusion in comparing dose rates with others, a nominal maximum DR is listed as 600MU/min, per Elekta guidance recommendation for entry into the TPS. Actual DRs for our LINAC, displayed during delivery, are given in Table 4 and may vary several percent during treatment day. Flatness and symmetry for the beams at a depth of 10 cm are within specifications for 600 MU/min DR, measured using a water tank. The 2D diode array relative results show crossbeam profile results within 2% of the 600 MU/min value for 6 MV vertical gantry and 358° arc (Figs. 2 and 3) with a comparison between the vertical, horizontal, and arc (Fig. 4) for 600 MU/min. Only these results are shown, but all 6 and 10 MV measurements (vertical gantry, horizontal gantry, and 358° arc) for both in‐plane and cross‐plane show agreement with the 600 MU/min value within 2% with an occasional 3% diode at the lowest or next‐lowest DR. These results, while similar in trend to Bedford and Warrington,^(7)^ demonstrate a smaller difference between the DRs even with the addition of ${\left(\Delta \text{MU}/\Delta t\right)}_{\max}/32$ data.

**Table 4:** Binned DRs of the linac tested. Nominal DRs are suggested for entry into the TPS, and are used in the text and figures.

*Binned DR*	*Nominal DR (MU/min)*	*6 MV Actual DR (MU/min)*	*10 MV Actual DR (MU/min)*
Max	600	435	475
Max/2	300	216	236
Max/4	150	108	118
Max/8	75	53	59
Max/16	38	27	30
Max/32	19	12	14

#### B.1 Dynamic MLC positional tests

##### B.1.1 Picket Fence test for static gantry cardinals and arc

**Figure 5:**
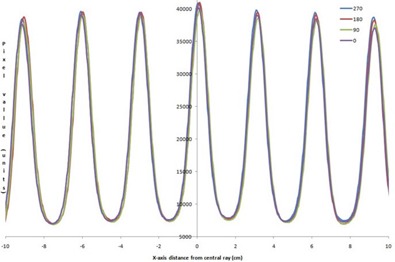
Picket Fence positions for stationary cardinal angles. Gantry angles are noted in the legend.

**Figure 6:**
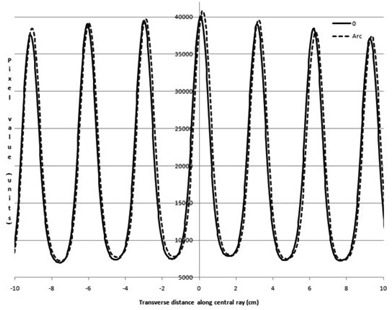
Picket Fence for vertical gantry and 356° angle arc.

MLC positional verification of dynamic MLC Picket Fences was measured using the EPID (Figs. 5 and 6). Peaks were identified by finding the local pixel having the maximum density value, which for these measurements, was always unique. Peaks were within ±0.5mm and 1 mm for the stationary cardinal angles and 180° arc, respectively (Table 5).

**Table 5:** Picket Fence peak occurrence for cardinal angles and arc verifying MLC positioning accuracy.

	*Peak Occurrence (cm)*						
*Gantry 270*	*Gantry 180*	*Gantry 90*	*Gantry 0*	*Arc 356°*	*Static Range (cm)*	*Average Static (cm)*	*Arc ‐ Ave Static (cm)*
−9.16	−9.06	−9.08	−9.16	−9.08	0.102	−9.11	0.032
−6.06	−5.98	−5.96	−6.06	−5.98	0.102	−6.02	0.032
−3.01	−2.94	−2.94	−2.99	−2.91	0.077	−2.97	0.058
0.06	0.14	0.14	0.06	0.19	0.077	0.10	−0.090
3.11	3.16	3.19	3.13	3.21	0.077	3.15	−0.064
6.16	6.21	6.26	6.21	6.31	0.102	6.21	−0.102
9.20	9.25	9.31	9.25	9.36	0.102	9.25	−0.102

##### B.1.2 Picket Fence test with intentional errors

**Figure 7:**
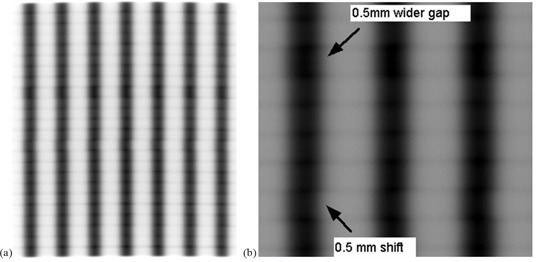
Picket Fence with intentional errors of 0.5 mm wider gap and 0.5 mm horizontal shift shown for: a) 25 cm by 25 cm field, and b) magnified portion.

The intentional errors in the Picket Fence arc were noticeable visually (Fig. 7(a) and (b)). Graphical analysis similar to Figs. 5 and 6 (not shown) also indicated the errors.

#### C.1. Maximum dynamic values

The ${\text{DR}}_{\max}$ values for 6 and 10 MV were within 1% of the displayed values, based on stopwatch measurements. For 500 MUs delivered to determine ${\text{DR}}_{\max}$, we found it easier to time the last 400 MUs as there was ramp‐up speed at initiation of the irradiation. An error of 0.5 sec in starting/stopping the stopwatch overlapped with perfect agreement. The maximum MLC speed, backup jaw speed, and gantry speed are less than 0.5% from values entered in the controlling software for 6 MV with somewhat larger differences for 10 MV (Table 6). These values were verified frequently prior to subsequent tests to ensure we were driving each parameter to the limit; small variations were seen between the values from day to day, but they were consistent.

**Table 6:** Maximum values of DR, MLC speed, backup jaw speed, and GS determined using internal consistency tests described in text.

	*DR Accuracy* ^a^	*MLC* ${\left(\Delta x/\Delta t\right)}_{\text{max}}\;\text{cm}/\text{sec}$	*Jaw* ${\left(\Delta x/\Delta t\right)}_{\text{max}}\;\text{cm}/\text{sec}$	*Gantry* ${\left(\Delta \theta /\Delta t\right)}_{\text{max}}{\;}^{\circ }/\text{sec}$
Nominal		2.0	1.5	6.0
6 MV	0.99	1.99	1.49	6.00
10 MV	0.99	1.94	1.46	5.90

a Displayed (MU/min)/Measured (MU/min); error of 0.5sec overlaps with 1.00

##### C.1.1 Controlling DR and GS

Tests controlling DR and GS show accurate delivery within ±1% was delivered at for 6 and 10 MV (Table 7) with standard deviations ranging from 0.4 to 1.7%. During initial 10X testing, the strip with ${\left(\Delta \text{MU}/\Delta t\right)}_{\max}:{\left(\Delta \theta /\Delta t\right)}_{\max}$ was delivered at ${\left(\Delta \text{MU}/\Delta t\right)}_{\max}/2:{\left(\Delta \theta /\Delta t\right)}_{\max}/2$. While the correct dose was delivered, we found that the strip would be delivered as planned with ${\left(\Delta \theta /\Delta t\right)}_{\max}$ of 5.86°/sec. The departure from 5.9°/sec, measured using a 90° arc, is likely due to the gantry speed ramp‐up time with the shorter arc used in this test (33°), which is controlled by the gantry inertia compensation distance parameter (which we do not calculate).

**Table 7:** Eight strips of 3 cm width with the same dose but delivered with different DR and GS. Doses were measured using an isocentrically‐mounted 2D diode array. Results are midstrip, and are normalized to open‐field measurements with the same DR.

(ΔMU/Δt)→	max/32	max/16	max/8	max/4	max/2	*max*	*1.2max*	*max*
(Δθ/Δt)→	max/32	max/16	max/8	max/4	max/2	*max*	*max*	*1.2max*
6 MV	99.0±0.6	99.3±0.9	99.4±0.9	99.9±0.7	100.5±0.4	101.0±0.6	100.9±0.4	99.9±0.9
10 MV	99.3±0.6	100.3±0.9	100.0±1.7	99.9±0.8	100.5±1.6	100.6±1.1	100.4±0.6	100.5±0.6

##### C.1.2 Control of MLC speed and DR

Initial tests controlling MLC leaf speed and DR show accurate delivery within ±3.5% with standard deviations on the same order of magnitude and occasionally larger. Dose to the 2D detector array was about 3.3 cGy, which is quite low. Cumulating the dose over repeated irradiations (up to 10) did not improve the results of the mean or standard deviation. We decided to repeat the test using the EPID which, while having less dose due to the larger source‐to‐detector distance, has increased resolution. A transverse profile of half an MLC width (0.5 cm) centered 0.5 cm superior to the central ray was used for the analysis, which minimized interleaf leakage considerations. The same method of normalizing the test measurement with the open beam profile per the initial test design was used, but measured using the EPID instead of the 2D array, for each DR. Some consideration was given over how many pixels to average over. The results were within ±2% tolerance with smoothing over 5 pixels (1.3 mm), but the standard deviation (estimated using the raw data) was quite large. Increasing the pixel averaging to 7, 9, or 11 improved the agreement between the different strips; there was much better agreement with increased pixels. The standard deviation of the raw data average over 7, 9, and 11 pixels was about the same, and was about half the value as that using 5 pixels. It was decided to smooth the data over a 7‐pixel moving average (1.8 mm at 100 cm SAD). Results showing the 7‐pixel average at the strip midpoint are shown in Table 8 and are less than 2%.

**Table 8:** Seven strips of 3 cm width with the same dose but delivered with different MLC speeds and DR, normalized to open field with the same DR.

(Δx/Δt)→	max/32	max/16	max/8	max/4	max/2	*max*	*1.2max*
(ΔMU/Δt)→	max/32	max/16	max/8	max/4	max/2	*max*	*1.2max*
6 MV	98.2±1.8	100.6±3.5	98.7±2.8	101.7±2.3	100.0±4.2	101.0±3.3	99.9±3.1

##### C.1.3 MLC reversals

**Figure 8:**
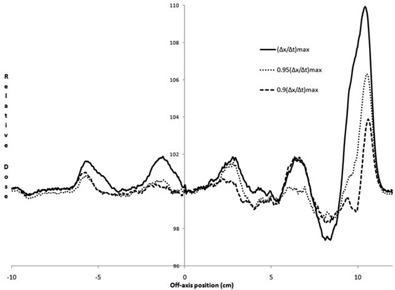
Normalized fluence when the MLCs are instantly reversed. The MLCs are moving with the pattern given in normalized with a similar irradiation having MLCs moving at 0.36 cm/sec, with single reversals centered at positions of −5.6, −1.6, 3.6, and 7.6 cm, and 4.5 reversals at 10.6 cm.

Normalized measurements of MLC reversals are given in Fig. 8. There is an increase of about 2%, 1.3%, and 1% fluence per reversal for 1.0, 0.95, 0.90 ${\left(\Delta x/\Delta t\right)}_{\max}$, respectively. Underdosing was 0.4, 0.2, and 0.1% for the same (Δx/Δt), respectively.

##### C.1.4 Dose‐rate changes

**Figure 9:**
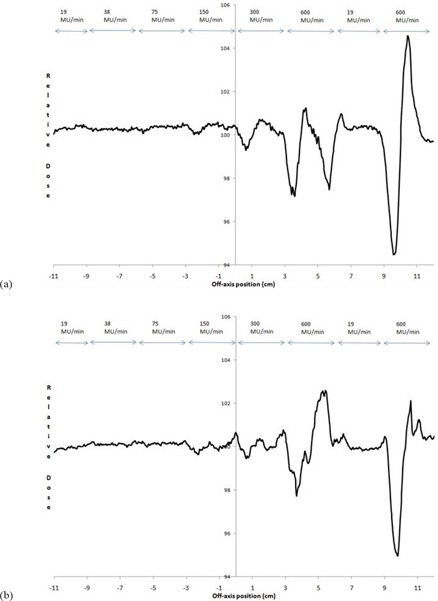
Normalized fluence when DRs were increased as the MLC bank moved from left to right in the a) 6 MV and b) 10 MV. Nominal dose rates for each strip are indicated. The test strip MLC speed was ${\left(\Delta x/\Delta t\right)}_{\max}/2\;(1\;\text{cm}/\sec)$, which was normalized using a similar irradiation having the same DRs and MLC speed of 0.25 cm/sec.

Differences of >2% were seen for 6 MV DR increases between ${\left(\Delta \text{MU}/\Delta t\right)}_{\max}/2\to {\left(\Delta \text{MU}/\Delta t\right)}_{\max}$, with a maximum of less than 3%. The extreme jump from ${\left(\Delta \text{MU}/\Delta t\right)}_{\max}/32\to {\left(\Delta \text{MU}/\Delta t\right)}_{\max}$ had doses >±3% over 9 mm of MLC leaf traversal (under‐ and overdose) (Fig. 9(a)). Results for 10 MV were similar to that for 6 MV (Fig. 9(b)), except the extreme change from ${\left(\Delta \text{MU}/\Delta t\right)}_{\max}/32\to {\left(\Delta \text{MU}/\Delta t\right)}_{\max}$ having doses >±3% had a smaller transverse width of about 6 mm, due to a negligible overdose portion.

**Figure 10:**
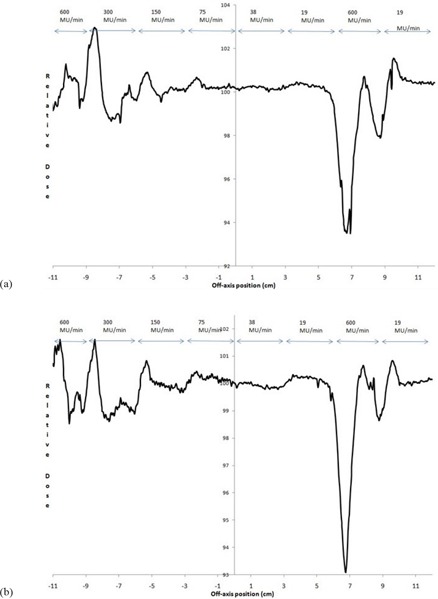
Normalized fluence when dose rates were decreased as the MLC bank moved from left to right in the a) 6 MV and b) 10 MV. Nominal dose rates for each strip are indicated. The test strip MLC speed was ${\left(\Delta x/\Delta t\right)}_{\max}/2\;(1\;\text{cm}/\sec)$, which was normalized using a similar irradiation having the same DRs and MLC speed of 0.25 cm/sec.

For DR decreases, 6 MV dose differences for ${\left(\Delta \text{MU}/\Delta t\right)}_{\max}\to {\left(\Delta \text{MU}/\Delta t\right)}_{\max}/2$ greater than 2% and 3% were seen over 6 mm and 2 mm transverse distance, respectively. The extreme case of ${\left(\Delta \text{MU}/\Delta t\right)}_{\max}\to {\left(\Delta \text{MU}/\Delta t\right)}_{\max}/32$ was within ±2% (Fig. 10(a)), which was unexpected as this same transition was needed for the DR increase test and showed a brief excursion greater than 2% (Fig. 9(a)). Dose differences for 10 MV DR decreases were within ±2%, even for the extreme case (Fig. 10(b)).

## IV. DISCUSSION

The differences between 6 and 10 MV in the physical parameters of MLC speed, backup‐jaw speed, and GS are measureable (Table 6), but not likely clinically relevant. While it is doubtful that these physical parameters vary with energy and more likely indicating systematic very small ${\left(\Delta \text{MU}/\Delta t\right)}_{\max}$ issues, the differences are not thought to be clinical significant. In hindsight, it is suggested in designing tests for each parameter to use 95% of the maximum value to avoid wasted time fixing nonclinically‐important findings such as a slightly lower GS (section C.1.1). This would be more efficient in allowing the same controlling files to be transported to additional LINACs. The tests should still include values beyond the maximum, as was done here to verify that the LINAC controller modifies values of MLC speed, DR, and GS needed to deliver the correct treatment.

In testing MLC speed accuracy, significant time was added due to the low signal that made measuring the dose with the 2D diode array problematic which, while improved with the EPID measurements, still had signal issues as discussed in Section C.1.2. A different test design is suggested with the strips having twice the DR than that given in the Methods and Materials Section above, which will double the dose: ${\left(\Delta x/\Delta t\right)}_{\max}/32:{\left(\Delta \text{MU}/\Delta t\right)}_{\max}/16$; ${\left(\Delta x/\Delta t\right)}_{\max}/16:{\left(\Delta \text{MU}/\Delta t\right)}_{\max}/8$; ${\left(\Delta x/\Delta t\right)}_{\max}/8:{\left(\Delta \text{MU}/\Delta t\right)}_{\max}/4$; ${\left(\Delta x/\Delta t\right)}_{\max}/4:{\left(\Delta \text{MU}/\Delta t\right)}_{\max}/2$; ${\left(\Delta x/\Delta t\right)}_{\max}/2:{\left(\Delta \text{MU}/\Delta t\right)}_{\max}$; ${\left(\Delta x/\Delta t\right)}_{\max}:{\left(\Delta \text{MU}/\Delta t\right)}_{\max}$ (see additional text for this strip and the 1.2x strip); $1.2{\left(\Delta x/\Delta t\right)}_{\max}:1.2{\left(\Delta \text{MU}/\Delta t\right)}_{\max}$. Note this configuration would result in the last two strips having half the dose of the previous strips, which would complicate the analysis. This problem could be solved with the MLCs reversing direction after the first traversal to give a traversal in the opposite direction for just the last two strips with the same parameter values during the first traversal. Based on results from Fig. 8, this single reversal will not add additional unplanned MLC‐reversal–caused fluence at the middle of a 3 cm wide strip.

The instant directional reversal of the MLCs was interesting (Fig. 8); and while our results based on clinically‐measured fluence differ with reported values,^(7)^ this is not considered clinically significant for many reasons. Rapid reversals are rare based on optimization considerations, and will not occur in the same locations in the patient while the gantry is rotating around —except, perhaps, in extreme cases, which hopefully will be caught in patient‐specific quality assurance tests. Rapid MLC reversal issues may become more significant as plan complexity increases (e.g., from prostate to pelvis with nodes), single arcs are favored over multiple arcs for efficiency sake, and leaf speed increases in the next generation of MLCs. To minimize commissioning time for busy physicists, it might be suggested that the rapid‐reversal test results could be consolidated with the revised MLC‐speed test discussed in the paragraph above; if the MLC speed test is within tolerance for the last strip that had a reversal, additional rapid reversal tests are not needed.

The DR change measurements (Figs. 9 and 10) have questionable clinical significance. The DR changes between binned values is reported to occur within 0.25 sec,^(7)^ which would bring under/overdosing seen in Figs. 9 and 10 back to unity within a transverse distance of 2.5 mm, which does not conflict with our results for DR changes between subsequent DR bins. We do not know presently if the excursion seen in the 32‐factor dose‐rate drop would be controlled better in clinical treatment mode. More practically, treatment planning excursions of this magnitude we have not seen at the console while running patient VMAT plans, although this may be a larger issue for more complex plans, and the DR is generally ${\left(\Delta \text{MU}/\Delta t\right)}_{\max}/2$ or less, which gives DR change issues in the 1% region. You could argue that the excursion would be worse if we used a MLC speed of 2 cm/sec instead of 1 cm/sec, which would increase the transverse distance of the unacceptable doses reported here; but clinically, the MLCs move slower in areas that require dose that also requires higher dose rates, and move faster in areas not requiring dose with lower dose rates — which minimizes the significance of these results. The vendor is actively working on having a continuously‐variable DR, instead of the current binned‐DR parameter, which may minimize this issue in the near future. The tests presented here could still be used after this is provided, except for verification of maximum GS, MLC speed, and jaw speed which require the tester to see the drop in the dose rate by a factor of 2.

## V. CONCLUSIONS

Commissioning VMAT for an Elekta linac using procedures given for RapidArc was carried out,^(6)^ in addition to several tests suggested for an Elekta linac.^(7)^ The results given here indicate that the Elekta linac can accurately deliver VMAT plans. While the iComCAT program we used is not for clinical treatment, it is assumed that a clinically‐approved program providing data to the linac will be no worse. Following these tests, TPS commissioning for VMAT needs to be carried out. It is suggested to use AAPM Task Group Report 119^(10)^ downloadable phantom structures and treatment planning objectives, with the VMAT results being compared to the IMRT results. Following this, retrospective mock‐patient VMAT studies can be carried out with comparisons to IMRT plans for the same patients.

Commissioning of VMAT should start with comprehensively testing the accurate operation of the linac, but must not be unduly burdensome for the busy clinical physicist. The testing suggested by Ling et al.^(6)^ has been carried out by the author and colleagues for RapidArc accelerators, within a time duration that seems appropriate. Several of the RapidArc linacs tested had problems requiring field engineering service, indicating the value of the tests. The tests suggested by Bedford and Warringtion^(7)^ for Elekta VMAT commissioning have important elements that are needed, especially DR issues, which we enhanced with arc measurements of beam profiles, quantitative fluence errors introduced due to MLC reversals as a function of number of reversals, and quantitative fluence errors introduced by DR changes. These enhancements were made by controlling the linac directly over the maximum ranges. The tests used here may catch errors that might be missed by relying on quality assurance testing of phantom plans alone.

## ACKNOWLEDGMENTS

The authors would like to acknowledge the kind assistance of Sergey Kulikov, MS, of Elekta, Ltd, for his demonstration on building iCOMCat files.

## References

[1] Yu CX . Intensity‐modulated arc therapy with dynamic multileaf collimator: an alternative to tomotheray. Phys Med Biol. 1995; 40 (9): 1435–49. 8532757 10.1088/0031-9155/40/9/004

[2] Shepard DM , Cao D , Afghan MK , Earl MA . An arc‐sequencing algorithm for intensity modulated arc therapy. Med Phys. 2007; 34 (2): 464–70. 17388162 10.1118/1.2409239

[3] Otto K . Volumetric modulated arc therapy: IMRT in a single gantry arc. Med Phys. 2008; 35 (1): 310–17. 18293586 10.1118/1.2818738

[4] Cao D , Holmes TW , Afghan MK , Shepard DM . Comparison of plan quality provided by intensity‐modulated arc therapy and helical tomotherapy. Int J Radiat Oncol Biol Phy. 2007; 69 (1): 240–50. 10.1016/j.ijrobp.2007.04.073 17707278

[5] Carol M , Grant WH 3rd , Pavord D , et al. Initial clinical experience with Peacock intensity modulation of a 3‐D conformal radiation therapy system. Stereotact Funct Neurosurg. 1996; 66 (1–3): 30–34. 8938930 10.1159/000099664

[6] Ling CC , Zhang P , Archambault Y , Bocanek J , Tang G , LoSasso T . Commissioning and quality assurance of RapidArc radiotherapy delivery system. Int J Radiat Oncol Biol Phys. 2008; 72 (2): 575–81. 18793960 10.1016/j.ijrobp.2008.05.060

[7] Bedford JL and Warrington AP . Commissioning of volumetric modulated arc therapy (VMAT). Int J Radiat Oncol Biol Phys. 2009; 73 (2): 537–45. 19147018 10.1016/j.ijrobp.2008.08.055

[8] Ezzell GA , Galvin JM , Low D , et al. Guidance document on delivery, treatment planning and clinical implementation of IMRT: report of the IMRT Subcommittee of the AAPM Radiation Therapy Committee. Med Phys. 2003; 30 (8): 2089–115. 12945975 10.1118/1.1591194

[9] Sastre‐Padro M , Lervag C , Eilertsen K , Malinen E . The performance of multileaf collimators evaluated by the stripe test. Med Dosim. 2009; 34 (3): 202–06. 19647629 10.1016/j.meddos.2008.08.005

[10] Ezzell GA , Burmeister JW , Dogan N , et al. IMRT commissioning: multiple institution planning and doismetry comparison, a report from AAPM Task Group 119. Med Phys. 2009; 36 (11): 5359–73. 19994544 10.1118/1.3238104

